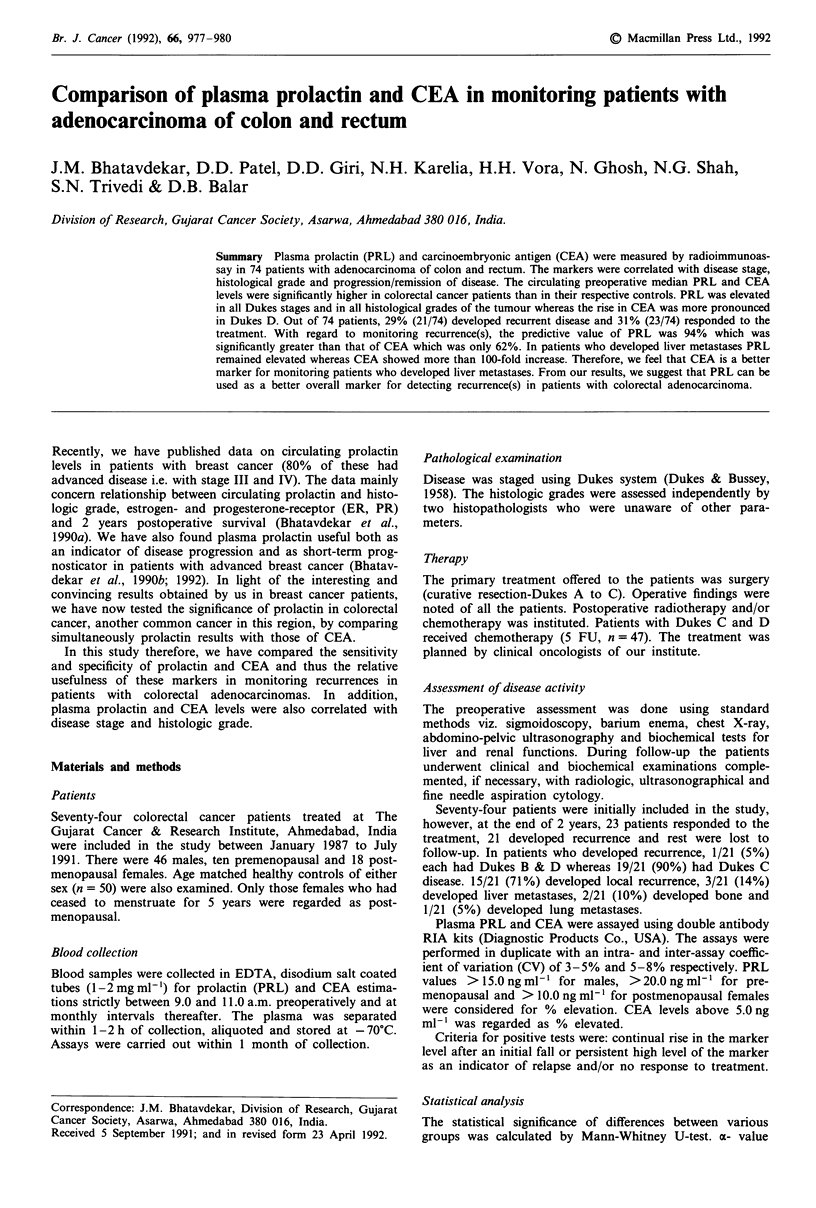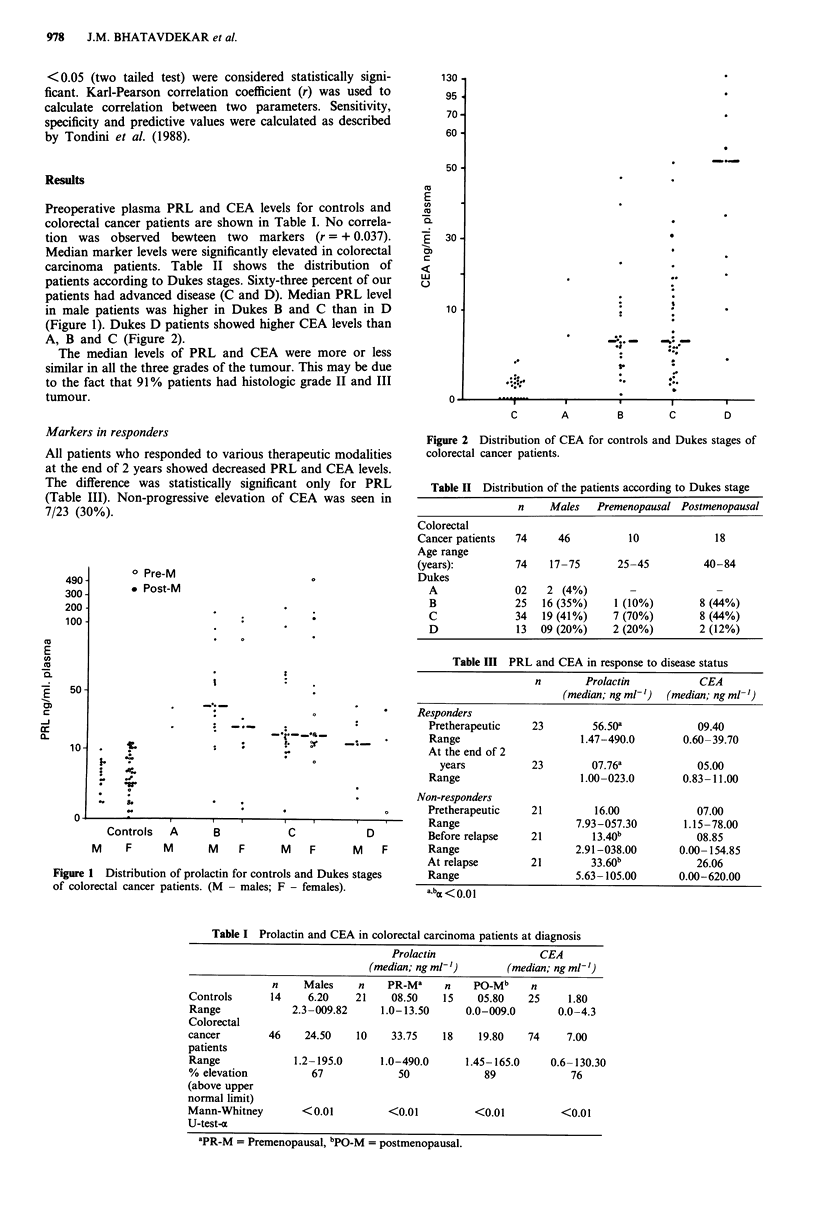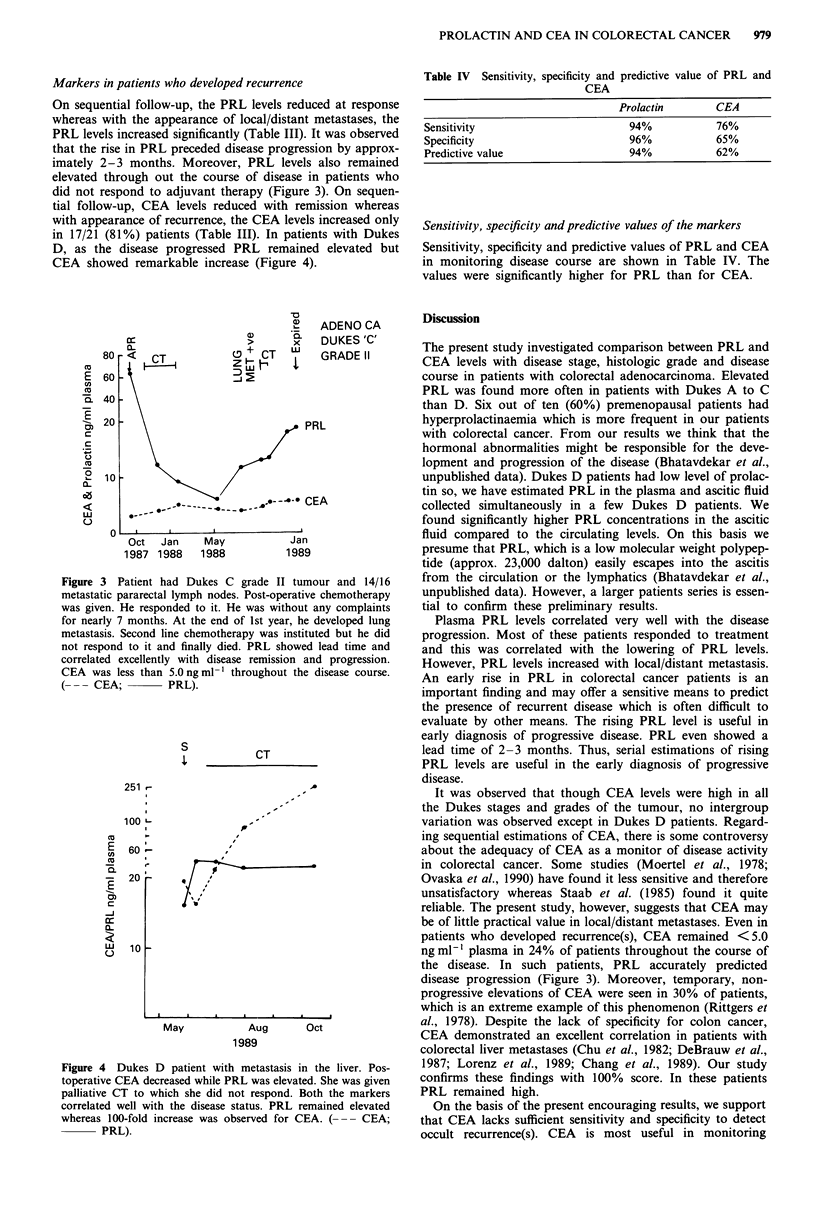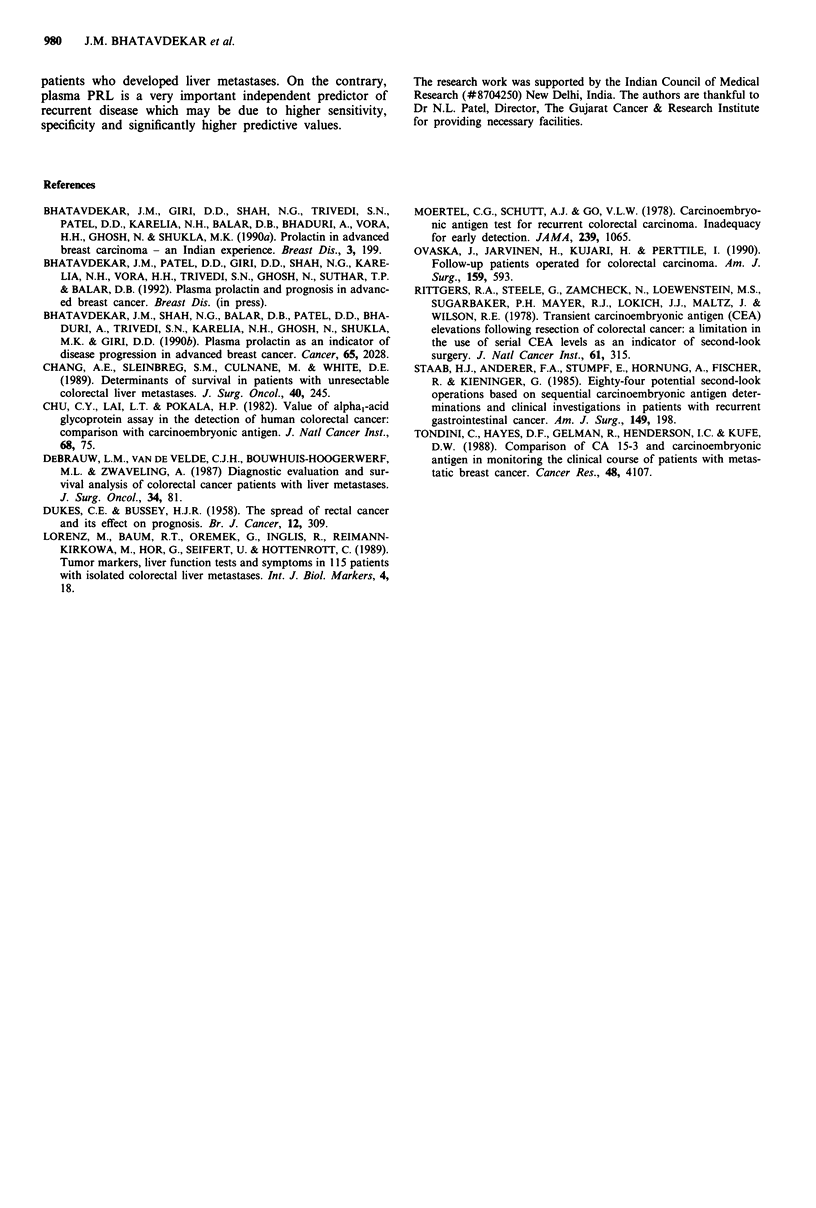# Comparison of plasma prolactin and CEA in monitoring patients with adenocarcinoma of colon and rectum.

**DOI:** 10.1038/bjc.1992.395

**Published:** 1992-11

**Authors:** J. M. Bhatavdekar, D. D. Patel, D. D. Giri, N. H. Karelia, H. H. Vora, N. Ghosh, N. G. Shah, S. N. Trivedi, D. B. Balar

**Affiliations:** Division of Research, Gujarat Cancer Society, Asarwa, Ahmedabad, India.

## Abstract

Plasma prolactin (PRL) and carcinoembryonic antigen (CEA) were measured by radioimmunoassay in 74 patients with adenocarcinoma of colon and rectum. The markers were correlated with disease stage, histological grade and progression/remission of disease. The circulating preoperative median PRL and CEA levels were significantly higher in colorectal cancer patients than in their respective controls. PRL was elevated in all Dukes stages and in all histological grades of the tumour whereas the rise in CEA was more pronounced in Dukes D. Out of 74 patients, 29% (21/74) developed recurrent disease and 31% (23/74) responded to the treatment. With regard to monitoring recurrence(s), the predictive value of PRL was 94% which was significantly greater than that of CEA which was only 62%. In patients who developed liver metastases PRL remained elevated whereas CEA showed more than 100-fold increase. Therefore, we feel that CEA is a better marker for monitoring patients who developed liver metastases. From our results, we suggest that PRL can be used as a better overall marker for detecting recurrence(s) in patients with colorectal adenocarcinoma.


*Br. J. Cancer* (1992), **66**, 977–980 © Macmillan Press Ltd., 1992

### Comparison of plasma prolactin and CEA in monitoring patients with adenocarcinoma of colon and rectum

J.M. Bhatavdekar, D.D. Patel, D.D. Giri, N.H. Karelia, H.H. Vora, N. Ghosh, N.G. Shah, S.N. Trivedi & D.B. Balar

*Division of Research, Gujarat Cancer Society, Asarwa, Ahmedabad 380 016, India.*

**Summary** Plasma prolactin (PRL) and carcinoembryonic antigen (CEA) were measured by radioimmunoassay in 74 patients with adenocarcinoma of colon and rectum. The markers were correlated with disease stage, histological grade and progression/remission of disease. The circulating preoperative median PRL and CEA levels were significantly higher in colorectal cancer patients than in their respective controls. PRL was elevated in all Dukes stages and in all histological grades of the tumour whereas the rise in CEA was more pronounced in Dukes D. Out of 74 patients, 29% (21/74) developed recurrent disease and 31% (23/74) responded to the treatment. With regard to monitoring recurrence(s), the predictive value of PRL was 94% which was significantly greater than that of CEA which was only 62%. In patients who developed liver metastases PRL remained elevated whereas CEA showed more than 100-fold increase. Therefore, we feel that CEA is a better marker for monitoring patients who developed liver metastases. From our results, we suggest that PRL can be used as a better overall marker for detecting recurrence(s) in patients with colorectal adenocarcinoma.

Recently, we have published data on circulating prolactin levels in patients with breast cancer (80% of these had advanced disease i.e. with stage III and IV). The data mainly concern relationship between circulating prolactin and histologic grade, estrogen- and progesterone-receptor (ER, PR) and 2 years postoperative survival (Bhatavdekar *et al.*, 1990*a*). We have also found plasma prolactin useful both as an indicator of disease progression and as short-term prognosticator in patients with advanced breast cancer (Bhatavdekar *et al.*, 1990*b*; 1992). In light of the interesting and convincing results obtained by us in breast cancer patients, we have now tested the significance of prolactin in colorectal cancer, another common cancer in this region, by comparing simultaneously prolactin results with those of CEA.

In this study therefore, we have compared the sensitivity and specificity of prolactin and CEA and thus the relative usefulness of these markers in monitoring recurrences in patients with colorectal adenocarcinomas. In addition, plasma prolactin and CEA levels were also correlated with disease stage and histologic grade.

### Materials and methods

### *Patients*

Seventy-four colorectal cancer patients treated at The Gujarat Cancer & Research Institute, Ahmedabad, India were included in the study between January 1987 to July 1991. There were 46 males, ten premenopausal and 18 postmenopausal females. Age matched healthy controls of either sex ($n = 50$) were also examined. Only those females who had ceased to menstruate for 5 years were regarded as postmenopausal.

### *Blood collection*

Blood samples were collected in EDTA, disodium salt coated tubes ($1-2\ mg\ ml^{-1}$) for prolactin (PRL) and CEA estimations strictly between 9.0 and 11.0 a.m. preoperatively and at monthly intervals thereafter. The plasma was separated within 1–2 h of collection, aliquoted and stored at $-70°C$. Assays were carried out within 1 month of collection.

Correspondence: J.M. Bhatavdekar, Division of Research, Gujarat Cancer Society, Asarwa, Ahmedabad 380 016, India.
Received 5 September 1991; and in revised form 23 April 1992.

### *Pathological examination*

Disease was staged using Dukes system (Dukes & Bussey, 1958). The histologic grades were assessed independently by two histopathologists who were unaware of other parameters.

### *Therapy*

The primary treatment offered to the patients was surgery (curative resection-Dukes A to C). Operative findings were noted of all the patients. Postoperative radiotherapy and/or chemotherapy was instituted. Patients with Dukes C and D received chemotherapy (5 FU, $n = 47$). The treatment was planned by clinical oncologists of our institute.

### *Assessment of disease activity*

The preoperative assessment was done using standard methods viz. sigmoidoscopy, barium enema, chest X-ray, abdomino-pelvic ultrasonography and biochemical tests for liver and renal functions. During follow-up the patients underwent clinical and biochemical examinations complemented, if necessary, with radiologic, ultrasonographical and fine needle aspiration cytology.

Seventy-four patients were initially included in the study, however, at the end of 2 years, 23 patients responded to the treatment, 21 developed recurrence and rest were lost to follow-up. In patients who developed recurrence, 1/21 (5%) each had Dukes B & D whereas 19/21 (90%) had Dukes C disease. 15/21 (71%) developed local recurrence, 3/21 (14%) developed liver metastases, 2/21 (10%) developed bone and 1/21 (5%) developed lung metastases.

Plasma PRL and CEA were assayed using double antibody RIA kits (Diagnostic Products Co., USA). The assays were performed in duplicate with an intra- and inter-assay coefficient of variation (CV) of 3–5% and 5–8% respectively. PRL values $>15.0\ ng\ ml^{-1}$ for males, $>20.0\ ng\ ml^{-1}$ for premenopausal and $>10.0\ ng\ ml^{-1}$ for postmenopausal females were considered for % elevation. CEA levels above $5.0\ ng\ ml^{-1}$ was regarded as % elevated.

Criteria for positive tests were: continual rise in the marker level after an initial fall or persistent high level of the marker as an indicator of relapse and/or no response to treatment.

### *Statistical analysis*

The statistical significance of differences between various groups was calculated by Mann-Whitney U-test. α- value

<0.05 (two tailed test) were considered statistically significant. Karl-Pearson correlation coefficient ($r$) was used to calculate correlation between two parameters. Sensitivity, specificity and predictive values were calculated as described by Tondini et al. (1988).

### Results

Preoperative plasma PRL and CEA levels for controls and colorectal cancer patients are shown in Table I. No correlation was observed bewteen two markers ($r = +0.037$). Median marker levels were significantly elevated in colorectal carcinoma patients. Table II shows the distribution of patients according to Dukes stages. Sixty-three percent of our patients had advanced disease (C and D). Median PRL level in male patients was higher in Dukes B and C than in D (Figure 1). Dukes D patients showed higher CEA levels than A, B and C (Figure 2).

The median levels of PRL and CEA were more or less similar in all the three grades of the tumour. This may be due to the fact that 91% patients had histologic grade II and III tumour.

### *Markers in responders*

All patients who responded to various therapeutic modalities at the end of 2 years showed decreased PRL and CEA levels. The difference was statistically significant only for PRL (Table III). Non-progressive elevation of CEA was seen in 7/23 (30%).

**Figure 1** Distribution of prolactin for controls and Dukes stages of colorectal cancer patients. (M – males; F – females).

**Figure 2** Distribution of CEA for controls and Dukes stages of colorectal cancer patients.

**Table II** Distribution of the patients according to Dukes stage

| | *n* | *Males* | *Premenopausal* | *Postmenopausal* |
|---|---|---|---|---|
| Colorectal Cancer patients | 74 | 46 | 10 | 18 |
| Age range (years): | 74 | 17–75 | 25–45 | 40–84 |
| Dukes | | | | |
| A | 02 | 2 (4%) | – | – |
| B | 25 | 16 (35%) | 1 (10%) | 8 (44%) |
| C | 34 | 19 (41%) | 7 (70%) | 8 (44%) |
| D | 13 | 09 (20%) | 2 (20%) | 2 (12%) |

**Table III** PRL and CEA in response to disease status

| | *n* | *Prolactin (median; ng ml$^{-1}$)* | *CEA (median; ng ml$^{-1}$)* |
|---|---|---|---|
| *Responders* | | | |
| Pretherapeutic | 23 | 56.50[a] | 09.40 |
| Range | | 1.47–490.0 | 0.60–39.70 |
| At the end of 2 years | 23 | 07.76[a] | 05.00 |
| Range | | 1.00–023.0 | 0.83–11.00 |
| *Non-responders* | | | |
| Pretherapeutic | 21 | 16.00 | 07.00 |
| Range | | 7.93–057.30 | 1.15–78.00 |
| Before relapse | 21 | 13.40[b] | 08.85 |
| Range | | 2.91–038.00 | 0.00–154.85 |
| At relapse | 21 | 33.60[b] | 26.06 |
| Range | | 5.63–105.00 | 0.00–620.00 |

[a,b]$\alpha < 0.01$

**Table I** Prolactin and CEA in colorectal carcinoma patients at diagnosis

| | | *Prolactin (median; ng ml$^{-1}$)* | | | | | *CEA (median; ng ml$^{-1}$)* | |
|---|---|---|---|---|---|---|---|---|
| | *n* | Males | *n* | PR-M[a] | *n* | PO-M[b] | *n* | |
| Controls | 14 | 6.20 | 21 | 08.50 | 15 | 05.80 | 25 | 1.80 |
| Range | | 2.3–009.82 | | 1.0–13.50 | | 0.0–009.0 | | 0.0–4.3 |
| Colorectal cancer patients | 46 | 24.50 | 10 | 33.75 | 18 | 19.80 | 74 | 7.00 |
| Range | | 1.2–195.0 | | 1.0–490.0 | | 1.45–165.0 | | 0.6–130.30 |
| % elevation (above upper normal limit) | | 67 | | 50 | | 89 | | 76 |
| Mann-Whitney U-test-α | | <0.01 | | <0.01 | | <0.01 | | <0.01 |

[a]PR-M = Premenopausal, [b]PO-M = postmenopausal.

*Markers in patients who developed recurrence*

On sequential follow-up, the PRL levels reduced at response whereas with the appearance of local/distant metastases, the PRL levels increased significantly (Table III). It was observed that the rise in PRL preceded disease progression by approximately 2–3 months. Moreover, PRL levels also remained elevated through out the course of disease in patients who did not respond to adjuvant therapy (Figure 3). On sequential follow-up, CEA levels reduced with remission whereas with appearance of recurrence, the CEA levels increased only in 17/21 (81%) patients (Table III). In patients with Dukes D, as the disease progressed PRL remained elevated but CEA showed remarkable increase (Figure 4).

**Figure 3** Patient had Dukes C grade II tumour and 14/16 metastatic pararectal lymph nodes. Post-operative chemotherapy was given. He responded to it. He was without any complaints for nearly 7 months. At the end of 1st year, he developed lung metastasis. Second line chemotherapy was instituted but he did not respond to it and finally died. PRL showed lead time and correlated excellently with disease remission and progression. CEA was less than 5.0 ng ml$^{-1}$ throughout the disease course. (--- CEA; —— PRL).

**Figure 4** Dukes D patient with metastasis in the liver. Postoperative CEA decreased while PRL was elevated. She was given palliative CT to which she did not respond. Both the markers correlated well with the disease status. PRL remained elevated whereas 100-fold increase was observed for CEA. (--- CEA; —— PRL).

**Table IV** Sensitivity, specificity and predictive value of PRL and CEA

| | *Prolactin* | *CEA* |
|---|---|---|
| Sensitivity | 94% | 76% |
| Specificity | 96% | 65% |
| Predictive value | 94% | 62% |

*Sensitivity, specificity and predictive values of the markers*

Sensitivity, specificity and predictive values of PRL and CEA in monitoring disease course are shown in Table IV. The values were significantly higher for PRL than for CEA.

### Discussion

The present study investigated comparison between PRL and CEA levels with disease stage, histologic grade and disease course in patients with colorectal adenocarcinoma. Elevated PRL was found more often in patients with Dukes A to C than D. Six out of ten (60%) premenopausal patients had hyperprolactinaemia which is more frequent in our patients with colorectal cancer. From our results we think that the hormonal abnormalities might be responsible for the development and progression of the disease (Bhatavdekar *et al.*, unpublished data). Dukes D patients had low level of prolactin so, we have estimated PRL in the plasma and ascitic fluid collected simultaneously in a few Dukes D patients. We found significantly higher PRL concentrations in the ascitic fluid compared to the circulating levels. On this basis we presume that PRL, which is a low molecular weight polypeptide (approx. 23,000 dalton) easily escapes into the ascitis from the circulation or the lymphatics (Bhatavdekar *et al.*, unpublished data). However, a larger patients series is essential to confirm these preliminary results.

Plasma PRL levels correlated very well with the disease progression. Most of these patients responded to treatment and this was correlated with the lowering of PRL levels. However, PRL levels increased with local/distant metastasis. An early rise in PRL in colorectal cancer patients is an important finding and may offer a sensitive means to predict the presence of recurrent disease which is often difficult to evaluate by other means. The rising PRL level is useful in early diagnosis of progressive disease. PRL even showed a lead time of 2–3 months. Thus, serial estimations of rising PRL levels are useful in the early diagnosis of progressive disease.

It was observed that though CEA levels were high in all the Dukes stages and grades of the tumour, no intergroup variation was observed except in Dukes D patients. Regarding sequential estimations of CEA, there is some controversy about the adequacy of CEA as a monitor of disease activity in colorectal cancer. Some studies (Moertel *et al.*, 1978; Ovaska *et al.*, 1990) have found it less sensitive and therefore unsatisfactory whereas Staab *et al.* (1985) found it quite reliable. The present study, however, suggests that CEA may be of little practical value in local/distant metastases. Even in patients who developed recurrence(s), CEA remained $<5.0$ ng ml$^{-1}$ plasma in 24% of patients throughout the course of the disease. In such patients, PRL accurately predicted disease progression (Figure 3). Moreover, temporary, non-progressive elevations of CEA were seen in 30% of patients, which is an extreme example of this phenomenon (Rittgers *et al.*, 1978). Despite the lack of specificity for colon cancer, CEA demonstrated an excellent correlation in patients with colorectal liver metastases (Chu *et al.*, 1982; DeBrauw *et al.*, 1987; Lorenz *et al.*, 1989; Chang *et al.*, 1989). Our study confirms these findings with 100% score. In these patients PRL remained high.

On the basis of the present encouraging results, we support that CEA lacks sufficient sensitivity and specificity to detect occult recurrence(s). CEA is most useful in monitoring patients who developed liver metastases. On the contrary, plasma PRL is a very important independent predictor of recurrent disease which may be due to higher sensitivity, specificity and significantly higher predictive values.

The research work was supported by the Indian Council of Medical Research (#8704250) New Delhi, India. The authors are thankful to Dr N.L. Patel, Director, The Gujarat Cancer & Research Institute for providing necessary facilities.

### References

BHATAVDEKAR, J.M., GIRI, D.D., SHAH, N.G., TRIVEDI, S.N., PATEL, D.D., KARELIA, N.H., BALAR, D.B., BHADURI, A., VORA, H.H., GHOSH, N. & SHUKLA, M.K. (1990*a*). Prolactin in advanced breast carcinoma – an Indian experience. *Breast Dis.*, **3**, 199.

BHATAVDEKAR, J.M., PATEL, D.D., GIRI, D.D., SHAH, N.G., KARELIA, N.H., VORA, H.H., TRIVEDI, S.N., GHOSH, N., SUTHAR, T.P. & BALAR, D.B. (1992). Plasma prolactin and prognosis in advanced breast cancer. *Breast Dis.* (in press).

BHATAVDEKAR, J.M., SHAH, N.G., BALAR, D.B., PATEL, D.D., BHADURI, A., TRIVEDI, S.N., KARELIA, N.H., GHOSH, N., SHUKLA, M.K. & GIRI, D.D. (1990*b*). Plasma prolactin as an indicator of disease progression in advanced breast cancer. *Cancer*, **65**, 2028.

CHANG, A.E., SLEINBREG, S.M., CULNANE, M. & WHITE, D.E. (1989). Determinants of survival in patients with unresectable colorectal liver metastases. *J. Surg. Oncol.*, **40**, 245.

CHU, C.Y., LAI, L.T. & POKALA, H.P. (1982). Value of alpha$_1$-acid glycoprotein assay in the detection of human colorectal cancer: comparison with carcinoembryonic antigen. *J. Natl Cancer Inst.*, **68**, 75.

DEBRAUW, L.M., VAN DE VELDE, C.J.H., BOUWHUIS-HOOGERWERF, M.L. & ZWAVELING, A. (1987) Diagnostic evaluation and survival analysis of colorectal cancer patients with liver metastases. *J. Surg. Oncol.*, **34**, 81.

DUKES, C.E. & BUSSEY, H.J.R. (1958). The spread of rectal cancer and its effect on prognosis. *Br. J. Cancer*, **12**, 309.

LORENZ, M., BAUM, R.T., OREMEK, G., INGLIS, R., REIMANN-KIRKOWA, M., HOR, G., SEIFERT, U. & HOTTENROTT, C. (1989). Tumor markers, liver function tests and symptoms in 115 patients with isolated colorectal liver metastases. *Int. J. Biol. Markers*, **4**, 18.

MOERTEL, C.G., SCHUTT, A.J. & GO, V.L.W. (1978). Carcinoembryonic antigen test for recurrent colorectal carcinoma. Inadequacy for early detection. *JAMA*, **239**, 1065.

OVASKA, J., JARVINEN, H., KUJARI, H. & PERTTILE, I. (1990). Follow-up patients operated for colorectal carcinoma. *Am. J. Surg.*, **159**, 593.

RITTGERS, R.A., STEELE, G., ZAMCHECK, N., LOEWENSTEIN, M.S., SUGARBAKER, P.H. MAYER, R.J., LOKICH, J.J., MALTZ, J. & WILSON, R.E. (1978). Transient carcinoembryonic antigen (CEA) elevations following resection of colorectal cancer: a limitation in the use of serial CEA levels as an indicator of second-look surgery. *J. Natl Cancer Inst.*, **61**, 315.

STAAB, H.J., ANDERER, F.A., STUMPF, E., HORNUNG, A., FISCHER, R. & KIENINGER, G. (1985). Eighty-four potential second-look operations based on sequential carcinoembryonic antigen determinations and clinical investigations in patients with recurrent gastrointestinal cancer. *Am. J. Surg.*, **149**, 198.

TONDINI, C., HAYES, D.F., GELMAN, R., HENDERSON, I.C. & KUFE, D.W. (1988). Comparison of CA 15-3 and carcinoembryonic antigen in monitoring the clinical course of patients with metastatic breast cancer. *Cancer Res.*, **48**, 4107.